# Surface resistance to SSVs and SIRVs in pilin deletions of *Sulfolobus islandicus*

**DOI:** 10.1111/mmi.14435

**Published:** 2019-12-19

**Authors:** Elizabeth F. Rowland, Maria A. Bautista, Changyi Zhang, Rachel J. Whitaker

**Affiliations:** ^1^ Department of Microbiology University of Illinois at Urbana‐Champaign Urbana IL USA; ^2^ Department of Biological Sciences University of Calgary Calgary AB Canada; ^3^ Carl R. Woese Institute for Genomic Biology University of Illinois at Urbana‐Champaign Urbana IL USA

**Keywords:** archaea, pilin, resistance, S‐layer, SIRV, SSV, *Sulfolobus islandicus*, virus

## Abstract

Characterizing the molecular interactions of viruses in natural microbial populations offers insights into virus–host dynamics in complex ecosystems. We identify the resistance of *Sulfolobus islandicus* to *Sulfolobus* spindle‐shaped virus (SSV9) conferred by chromosomal deletions of pilin genes, *pilA1* and *pilA2* that are individually able to complement resistance. Mutants with deletions of both *pilA1* and *pilA2* or the prepilin peptidase, PibD, show the reduction in the number of pilins observed in TEM and reduced surface adherence but still adsorb SSV9. The proteinaceous outer S‐layer proteins, SlaA and SlaB, are not required for adsorption nor infection demonstrating that the S‐layer is not the primary receptor for SSV9 surface binding. Strains lacking both pilins are resistant to a broad panel of SSVs as well as a panel of unrelated *S. islandicus* rod‐shaped viruses (SIRVs). Unlike SSV9, we show that *pilA1* or *pilA2* is required for SIRV8 adsorption. In sequenced *Sulfolobus* strains from around the globe, one copy of each *pilA1* and *pilA2* is maintained and show codon‐level diversification, demonstrating their importance in nature. By characterizing the molecular interactions at the initiation of infection between *S. islandicus* and two different types of viruses we hope to increase the understanding of virus–host interactions in the archaeal domain.

## Graphical abstract

Evolving SSV9‐resistant *Sulfolobus islandicus* revealed pilin deletions in host chromosomes that confer broad viral resistance. Pilins were found to be a point of adsorption for *Sulfolobus* rod‐shaped virus 8, while SSV9 was still able to adsorb, but not infect. Furthermore, these pilins were found to be involved in surface adhesion as well as highly conserved and diversifying in all *Sulfolobus* genomes stressing their importance in the natural environment.

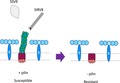

## 1 INTRODUCTION

A diverse array of novel viruses that infect the thermoacidophilic crenarchaea has been described (Peng, Garrett, & She, 2012; Prangishvili et al., 2017; Prangishvili & Garrett, 2005; Rice et al., 2001; Snyder, Bolduc, & Young, 2015). We do not yet understand the diversity and specificity of surface interactions involved in viral adsorption and entry, or how variation in surface structures may impact host ranges, susceptibility or dynamics in natural populations. The development of genetic tools in model crenarchaeon *Sulfolobus islandicus* has made it possible to identify and characterize the interactions between these viruses and their archaeal hosts (Zhang, Cooper, Krause, & Whitaker, 2013; Zhang, Phillips, Wipfler, Olsen, & Whitaker, 2018; Zhang & Whitaker, 2012, 2018).

Interactions with the cell surface form the first steps in viral attachment and entry. The predominant *Sulfolobales* surface structure is a proteinaceous, crystalline S‐layer that is comprised of two highly glycosylated proteins, SlaA and SlaB, that have been isolated and structurally analyzed by electron microscopy (Veith et al., 2009; Zhang et al., 2019). In addition, type IV pilus‐like structures are present and serve a diverse array of functions in *Sulfolobus* such as adherence and biofilm formation, cell‐to‐cell interactions, nutrient uptake, DNA transfer and facilitation of viral infection (Albers & Pohlschröder, 2009; Esquivel & Pohlschroder, 2014; Esquivel, Xu, & Pohlschroder, 2013; Henche, Koerdt, Ghosh, & Albers, 2012; Pohlschroder & Albers, 2016; Quemin et al., 2013; Silverman, 1997). The best characterized archaeal surface structure is the archaellum, which unlike the bacterial flagellum has structural similarities to a type IV‐pilus and contains a motor that rotates for swimming motility (Shahapure, Driessen, Haurat, Albers, & Dame, 2014). Minimally, all type IV pili including the archaellum are comprised of biosynthesis machinery including an ATPase (PilB), multi‐spanning membrane protein (PilC) and the pilin subunits themselves (PilA). Archaea generally carry multiple type IV pili loci in each genome (Wang et al., 2019) with these minimal components in a single operon (Albers & Pohlschröder, 2009; Esquivel et al., 2013; Makarova, Koonin, & Albers, 2016; Zolghadr, Klingl, Rachel, Driessen, & Albers, 2011). Pili structure and functions are all dependent on the enzyme PibD which is responsible for the maturation of the type IV prepilin subunits for the assembly of the pilus (Albers, Szabó, & Driessen, 2003; Szabó, Sani, et al., 2007; Szabó, Stahl, et al., 2007). Other type IV accessory genes are often present in the genome and can be essential for the function of the pilus (Makarova et al., 2016).

The hot spring environment of *S. islandicus* is a low complexity environment where viruses are the predominant predators and drivers of evolution (Bolduc, Wirth, Mazurie, & Young, 2015). Only a few of the diverse viruses in this environment have been characterized in molecular detail (Prangishvili et al., 2017). Here we focus on viruses infecting *S. islandicus, Sulfolobus* spindle‐shaped viruses (SSVs) and *S. islandicus* rod‐shaped virus (SIRVs), because they are the dominant plaque‐forming viruses in found in these natural populations (Pauly, Bautista, Black, & Whitaker, 2019).

SSVs are part of the *Fuselloviridae* family of viruses isolated from thermoacidophilic environments from hot springs worldwide. SSV particles contain circular, dsDNA genomes that persist episomally and are able to integrate into the tRNA genes of the host genome, resulting in chronic infection (Redder et al., 2009). Electron tomography data support a mechanism of release for the related virus, SSV1, whereby virus particle maturation occurs during budding from the host cell, as the particle is surrounded in a lipid layer and the S‐layer is condensed (Quemin et al., 2016). The S‐layer has been proposed as a possible receptor for SSVs cellular attachment based on the S‐layer structural lattice dimensions (Stedman, DeYoung, Saha, Sherman, & Morais, 2015). However, the specific mechanisms of SSVs interactions at the *S. islandicus* cell surface that lead to attachment and entry remain to be described.

SIRVs are members of the *Rudiviridae* family of viruses that infect *Sulfolobales.* These rod‐shaped viruses have a linear, dsDNA genome and release from their host through the formation of viral‐encoded pyramid structures formed at the cellular surface that lyse the host for virion release (Bize et al., 2009). Through electron microscopy, SIRV2 has been shown to interact with *S. islandicus* pili (Quemin et al., 2013). In addition, disruption of any of the four genes in *Sulfolobus solfataricus* P2 cells leads to SIRV2 resistance (Deng et al., 2014). Two of these genes are uncharacterized membrane‐associated proteins (SSO3139, SSO3140). The remaining two are homologous to type IV Archaeal adhesion pili (*aap*), AapE (PilB) and AapF (PilC) (SSO2387 and SSO2386 respectively) that have been characterized in *S. acidocaldarius* (Deng et al., 2014; Henche, Ghosh, et al., 2012).

In this study, we set out to experimentally identify virus–host surface interactions without a priori knowledge through a forward genetic screen in *S. islandicus*.

## 2 RESULTS

We evolved and isolated *S. islandicus* strains resistant to SSV9. In order to do this, we needed to remove the native CRISPR‐Cas immunity to SSV9 in our type strain RJW002 by deleting the CRISPR‐Cas array containing a spacer match to create strain ΔA1 (Bautista, Zhang, & Whitaker, 2015). In four independent experiments, ΔA1 was challenged with SSV9 at an multiplicity of infection (MOI) of 0.01 and SSV9‐resistant strains were screened for infection by PCR amplifying the SSV9 capsid gene (*vp2*) (Table S1). A single uninfected isolate from each experiment was confirmed to be resistant to SSV9 infection by a spot‐on‐lawn test. The four uninfected resistant *S. islandicus* genomes revealed converging chromosomal deletions ranging from 2 to 14 kb (Table S2). The minimal deletion region disrupts or deletes three genes (*M164_2742, M164_2745* and *M164_2746*) and an insertion element (*M164_2743* and *M164_2744*) in *S. islandicus* ΔA1 (Table S2).

**Figure 1:**
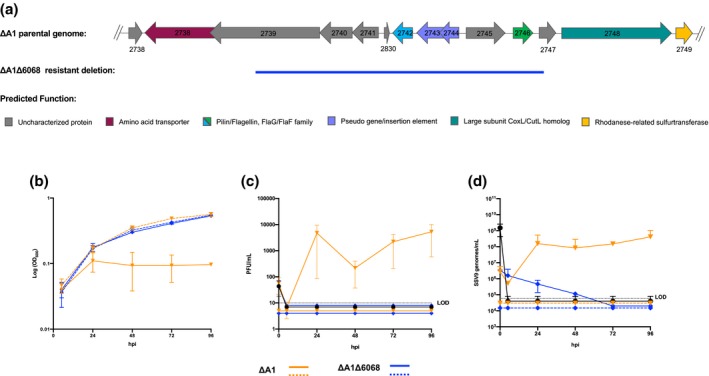
A 6068 bp chromosomal deletion prevents infection of *S. islandicus* by SSV9. (a) Gene cluster of the parental ΔA1 genome and the deleted or disrupted genes of the evolved resistant strain represented with a blue line. The arCOG predicted functions for the genes are color coded. (b) Host growth (OD_600_), (c) PFU/ml and (d) SSV9 genomes/ml over the course of SSV9 infection in ∆A1 (orange) and ∆A1∆6068 (blue). The strains challenged with SSV9 are represented in solid lines and the control lines are dashed. In c and d, SSV9 decay over the course of infection is represented (solid black)

The first resistant strain (∆A1.F6) contained a 6,068 bp chromosomal deletion as well as additional evolved mutations (Table S2). To isolate the phenotype of this 6,068 bp deletion, we used the previously described pop‐in/pop‐out method (Zhang et al., 2013; Zhang & Whitaker, 2012) to recreate the 6,068 bp deletion in the susceptible ancestor strain ΔA1, referred to herein as ΔA1Δ6068 (Figure 1a and Table S3). ΔA1Δ6068 was sequenced and compared to its ancestor (ΔA1) to confirm that no additional mutations were introduced (Table S3). The growth of ΔA1Δ6068 was compared to ΔA1 in the presence and absence of SSV9. (Figure 1b). Both strains exhibited the same growth in the absence of virus. However, while the ancestral ∆A1 strain showed growth inhibition and death in the presence of SSV9, the ∆A1∆6068 mutant was unaffected by the addition of SSV9 and exhibited growth similar to that seen in the absence of virus. In addition, when SSV9 was added to ∆A1 cells, virus multiplication was observed as both plaque forming units (PFU) (Figure 1c) and virus genome counts (Figure 1d) increased. This increase in virus numbers was not observed for the ∆A1∆6068 mutant culture (Figure 1c,d). Strain ΔA1Δ6068 was used for all future assays to characterize the conferred SSV9 resistance.

**Figure 2:**
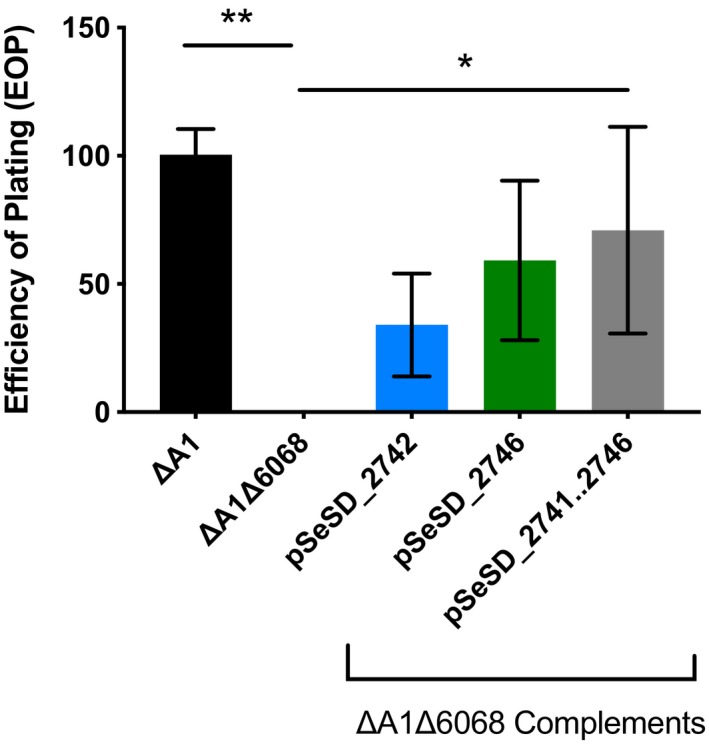
Complementation rescues infectability by SSV9. Infection assays measuring SSV9 infection (PFUs) in the immune deficient ancestor (ΔA1), resistant (ΔA1Δ6068) and resistant complemented with either *M164_2742*, *M164_2746* or *M164_2741…M164_2746*. Three biological replicate experiments measuring SSV9 infectivity of the resistant and complemented strains are shown with the average number of infected cells relative to the immune deficient ancestor (ΔA1). One‐way ANOVA was performed (*p < .05, **p < .005)

Homology searches using BLASTp (Altschul et al., 1997) revealed that two genes in the chromosomal deletion (*M164_2742* and *M164_2746*) have sequence similarity to the Archaeal Adhesive Pilins (*aap*) characterized in *S. acidocaldarius* (Figure S1, Henche, Ghosh, et al., 2012). To test whether deletions of the pilin genes resulted in resistance, a large region of the deletion (*M164_2741..2746*) and the genes *M164_2742* and *M164_2746* separately were cloned into a shuttle vector, pSeSD (Peng, Deng et al., 2012; Zhang et al., 2013; Zhang & Whitaker, 2012) under the control of their native promoters. Transformation of each of these plasmids into ΔA1Δ6068 resulted in increased sensitivity to SSV9 (Figures 2 and S2).

**Figure 3:**
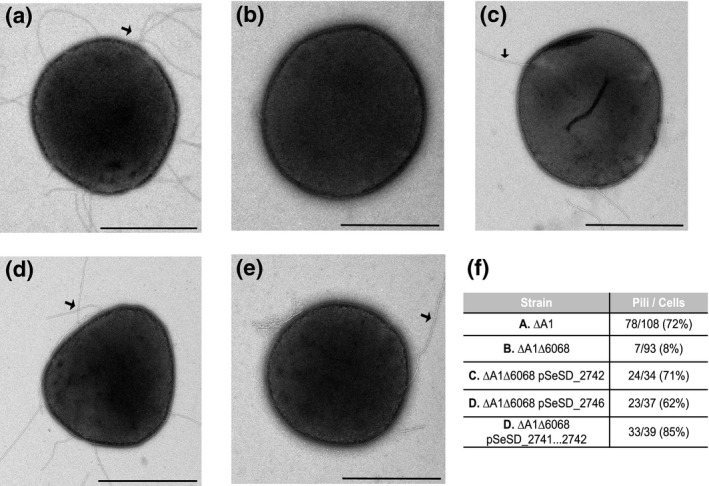
Genes within the deleted region encode pilins. Electron microscopy showing negative stained, whole cells of (a) SSV9‐susceptible parental strain, ∆A1 (b) Derived resistant, ∆A1∆6068. (c) ∆A1∆6068 pSeSD_2741…2746, (d) ∆A1∆6068 pSeSD_2742, (e) ∆A1∆6068 pSeSD_2746 and (f) Results of a double‐blind experiment in each strain where the number of cells containing pili observed over the total number of cells observed are reported. Scale bars are 1 µm. Black arrows indicate the examples of pilin structures

To establish whether *M164_2742* and *M164_2746* result in the formation of pili, we observed cells by transmission electron microscopy (TEM) (Figure 3). In double‐blinded experiments, 72% of the ΔA1 cells had pili (Figure 3a), while only 8% of the ΔA1Δ6068 cells had pili (Figure 3b). In the complemented strain, pSeSD_2741…2746 that includes both *M164_2742* and *M164_2746*, 85% of cells had pili (Figure 3e). When the coding and promoter sequences of either *M164_2742* or *M164_2746* were reintroduced into the ΔA1Δ6068 strain separately, 71% and 62% of the cells, respectively, had pili (Figure 3c,d). We note that there has been evidence that the expression of archaella is affected in other *Sulfolobus* species when either the pilins or pilin biosynthesis machinery is deleted (Henche, Koerdt, et al., 2012). However, for ΔA1Δ6068 as well as complemented strains we observed the same prevalence of archaella as for ∆A1 cells.

To test whether PibD processing and surface localization of *M164_2742* and *M164_2746* are necessary for SSV9 infection we constructed a marker‐insertion disruption strain, Δ*pibD::argD* (Figure S3a,b), in the pilus encoding *S. islandicus* strain, RJW004 (Zhang et al., 2013). When examining the resulting Δ*pibD::argD* strain under TEM, no pili or archaella were observed (Figure S3c). To test the susceptibility, we used an infectious SSV9 variant, SSV9.2, to evade CRISPR‐Cas immunity from the host background (See SSV9.2 supplemental file). When challenged with SSV9.2 at an MOI of 0.01, the Δ*pibD::argD* strain did not display any growth defect (Figure S3d), make additional viral particles in PFUs (Figure S3e), or replicate the viral genome as measured by qPCR (Figure S3f).

**Figure 4:**
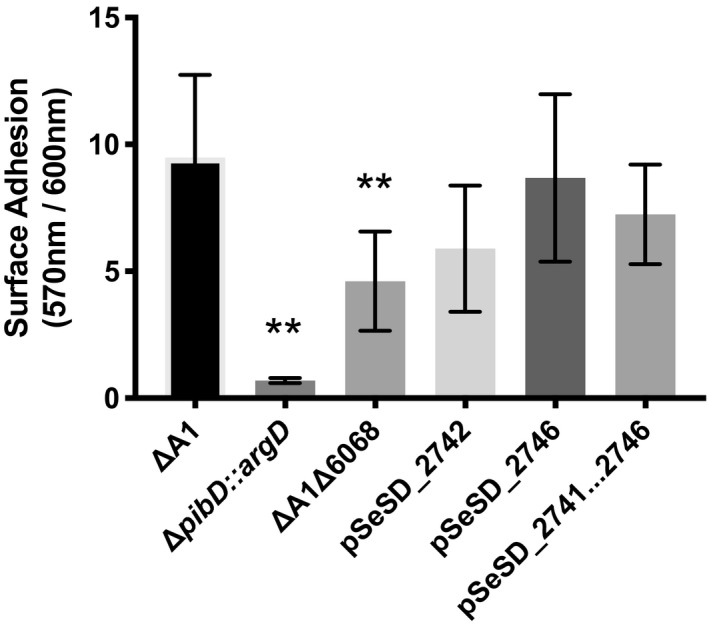
Comparison of biofilm formation between pilin containing ancestor strain, ∆A1 and pilin mutants. Microtiter assay was conducted measuring the crystal violet absorbance of attached cells (OD_570nm_) relative to planktonic cells (OD_600nm_) (Koerdt, Gödeke, Berger, Thormann, & Albers, 2010). Three biological replicates were assayed in triplicate. Significance was determined by one‐way ANOVA evaluating significance to ∆A1 (**p < .005)

Similar to the adhesion pilins (*aap*) described in *S. acidocaldarius* (Henche, Ghosh, et al., 2012), *M164_2742* and *M164_2746* are important for surface adhesion (Figures 4 and S4). When compared to *S. islandicus* RJW002 that contains *M164_2742* and *M164_2746*, ΔA1Δ6068 was less efficient in adhering to the glass coverslip. Coverslip adhesion was restored by complementation with either or both *M164_2742* and *M164_2746* (Figures 4 and S4). *S. islandicus* ΔA1Δ6068 adhered better than the adhesion deficient *S. islandicus *Δ*pibD::argD* strain, suggesting that other proteins processed by PibD are involved in effective surface adhesion (Esquivel et al., 2013; Henche, Koerdt, et al., 2012; Zolghadr et al., 2010). Based on homology and phenotypes, *M164_2742* and *M164_2746* are likely to be pilin genes. Therefore, *M164_2742* and *M164_2746* have been named *pilA1 and pilA2* respectively.

**Figure 5:**
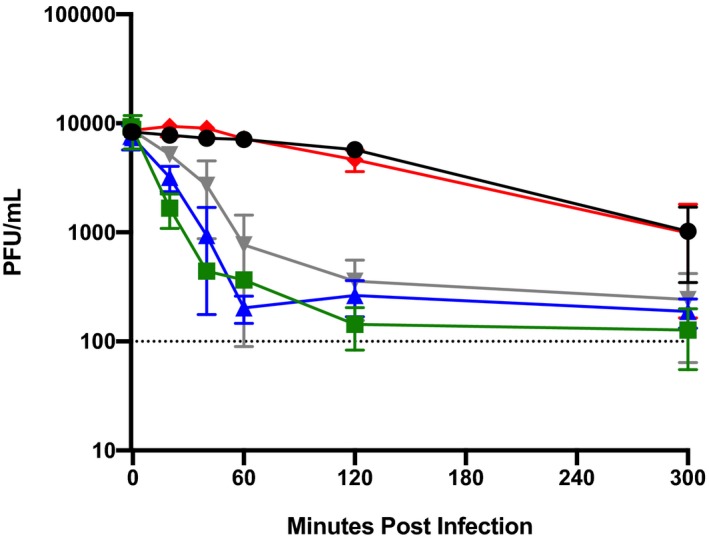
SSV9 adsorbs to host cells lacking pili. SSV9 adsorption was observed by measuring free virus particles over time (MOI = 0.001). The viral decay is shown (black) compared to the viral adsorption to RJW002 (green), ΔA1 Δ6068 (blue), *ΔpibD::argD* (gray) and *S. acidocaldarius* (red). *n* = 3

To test if pili provide a mechanism of attachment to the cellular envelope, we conducted adsorption assays with SSV9 (Bautista, Black, Youngblut, & Whitaker, 2017). Figure 5 shows that SSV9 adsorbs to ΔA1Δ6068, Δ*pibD::argD* in addition to RJW002 that contains *pilA1 and pilA2*, or the *pilA1* and *pilA2* complemented strains (data not shown). This result shows that, while *pilA1* and *pilA2* are necessary for infection by SSV9, neither appears to be required for adsorption. Adsorption was not observed with SSV9 to *S. acidocaldarius*, suggesting the specificity of the virus to PilA1 and PilA2 from *S. islandicus* (Figure 5).

**Figure 6:**
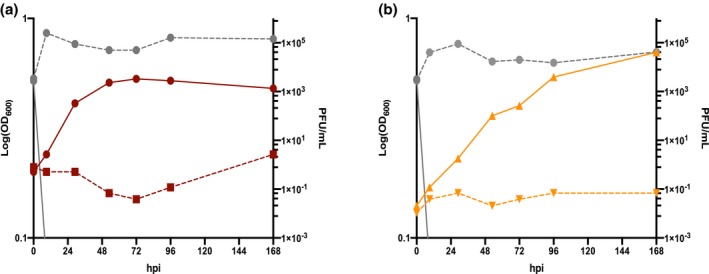
Δ*slaAB* can be productively infected by SSV9.2 virus. SSV9.2 infection of (a) RJW004 (red) and (b) Δ*slaAB* (orange). Host growth is measured (left axis) in the red (RJW004) and orange (Δ*slaAB*) lines. In both infections, the solid colored lines are the uninfected control and the SSV9.2 challenged cultures are dashed. The SSV9.2 PFU/ml was assayed from both culture supernatants in gray lines (right axis) from the control (solid) and the challenged (dashed) supernatants

Previous work has suggested that SSV1 adsorption requires the S‐layer in *S. solfataricus* P1 (Stedman et al., 2015; Zink et al., 2019). We have shown that *S. islandicus* lacking the outer S‐layer (SlaAB) is viable and contained pili and archaellum, although the archaellum are nonmotile in this strain (Zhang et al., 2019). The strain Δ*slaAB* also forms large aggregates in liquid culture (Zhang et al., 2019) preventing quantitative adsorption assays in this strain. To test whether S‐layer is necessary for productive infection (and indirectly adsorption), we challenged an S‐layer knockout strain Δ*slaAB* (Zhang et al., 2019) with SSV9.2. SSV9.2 was able to establish productive infections in both RJW004 (Figure 6a) and Δ*slaAB* (Figure 6b), supporting the idea that the *Sulfolobus* S‐layer does not play an essential role in adsorption or any other step in infection or viral release.

**Figure 7:**
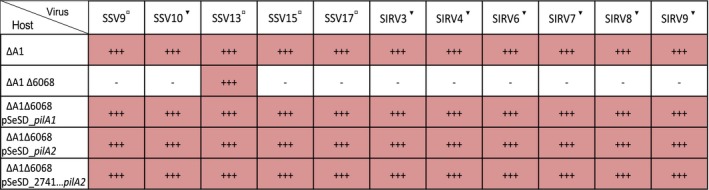
Pilin deletion confers resistance to a broad panel of viruses. Spots on lawn tests were performed with normalized virus titers to 1.47 × 10^3^ PFU/ml isolated from Kamchatka, Russia (▫︎) or Yellowstone National Park, USA (▾). Susceptibility to virus was tested with the susceptible strain: ∆A1, derived resistant strain: ∆A1∆6068, and plasmid complemented strains: ∆A1∆6068 pSeSD_*pilA1*, ∆A1 ∆6068 pSeSD_*pilA2* and ∆A1∆6068 pSeSD_2741…*pilA2*. Triplicate experiments are shown

Having shown that pilins encoded by *pilA1* and *pilA2* are necessary for infection, we tested the requirement for pili for susceptibility to SSVs and SIRVs isolated from multiple sources. The deletion in strain ΔA1Δ6068 provided resistance to other SSVs isolated from Kamchatka, Russia and Yellowstone National Park, USA, in addition to SSV9. Similarly, complementation by either *pilA1* or *pilA2* restored susceptibility to infection by these SSVs except SSV13. (Figure 7). In addition, a panel of unrelated SIRV viruses isolated from Yellowstone National Park, USA, (Bautista et al., 2017) was not able to infect ΔA1Δ6068 (Figure 7). Susceptibility to all SIRVs and SSVs was restored by either *pilA1* or *pilA2*. This broad viral resistance by the absence of *pilA1* and *pilA2* supports a common utilization of these pilins by SSVs and SIRVs for infection.

**Figure 8:**
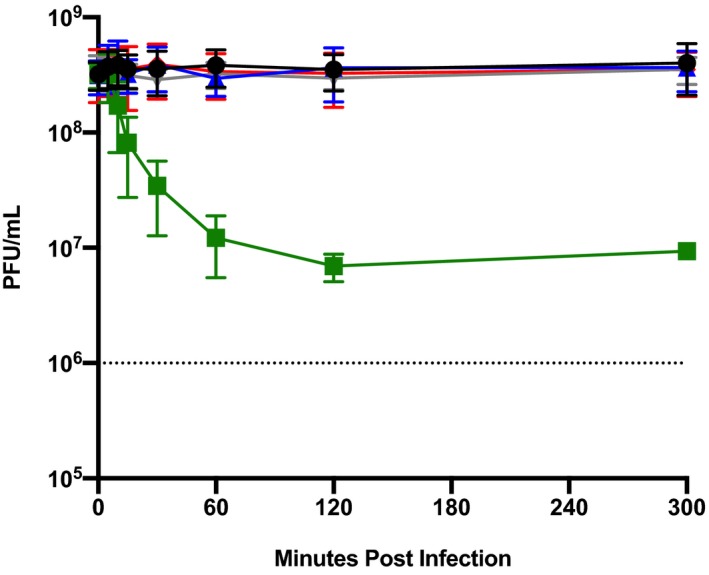
PilA1 and PilA2 are not required for SIRV8 adsorption. SIRV8 adsorption was done by measuring free virus particles over time (MOI = 1). The viral decay was shown (black) compared to the viral adsorption to RJW002 (green), ΔA1Δ6068 (blue), *ΔpibD::argD* (gray) and *S. acidocaldarius* (red). *n* = 3

To test the adsorption phenotype of SIRVs to ΔA1Δ6068, SIRV8 was used as a representative SIRV in adsorption assays. In contrast to SSV9, SIRV8 was unable to adsorb to the ΔA1Δ6068 or the pilin processing deficient Δ*pibD::argD* strain (Figure 8). Concentrated virus and host supernatants were visualized using TEM in an attempt to capture viral interactions with pili. We observed the interaction between pili and SIRV8, but not SSV9 (Figure S5).

**Figure 9:**
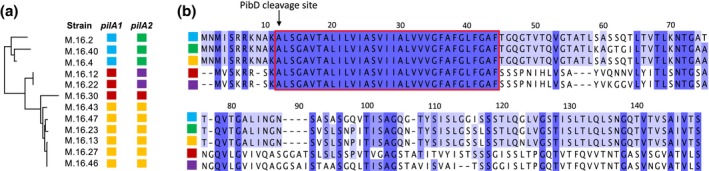
Five unique pilin genes are represented in the M.16 population. (a) Maximum‐likelihood tree of 12 *S. islandicus* genomes isolated from Kamchatka, Russia. Two pilins are present in each genome where each unique pilin sequence is represented in a different color. (b) Alignment of the five different pilin sequences' colors is correlated with their presence in each strain in (a). The proposed PibD cleavage site is denoted with arrow and the H‐domain is boxed in the red rectangle

Analyzing a cohort of 12 *S. islandicus* strain genomes (Cadillo‐Quiroz et al., 2012) revealed five unique but highly related alleles to *pilA1* and *pilA2* (Figure 9). Seven of the genomes contained two identical pilin genes (Figure 9a). However, all of the *pilA1* and *pilA2* genes were highly similar and the characteristic PibD cleavage site and the following H‐domain were conserved (Figure 9b). After examining all available *Sulfolobales* genomes, we found that each genome contains two copies of sequence homologs to these pilin genes, some encoded on the same strand, while others on opposite strands, even those isolated from the same hot springs as lytic SIRVs. The presence of *pilA1* and *pilA2* in every genome suggests that, while they are dispensable under controlled and media‐rich laboratory conditions, these pilins are likely essential for survival in nature. Comparison between sequences demonstrates a signature of diversifying selection at specific amino acid residues (Figure S1). This suggests that these positions in PilA1 and PilA2 may be under diversifying selection to evade lytic viruses.

## 3 DISCUSSION

We evolved CRISPR‐Cas‐independent resistance in *S. islandicus* strains derived from M.16.4 through the deletion of both copies of the type IV adhesive pilin genes *pilA1* and *pilA2*. Deletion of these two genes resulted in reduced pilus production and surface adherence. The absence of *pilA1* and *pilA2* also conferred resistance to infection by five of seven SSVs and six SIRVs isolated from different hot spring environments, suggesting that pili are important for infection with multiple viruses. In the case of SIRVs, loss of infectability is likely to result from the lack of adsorption but the same does not seem to be true for SSVs. The role of the pilin in SSV9 infection has yet to be determined.

The role that PilA1 and PilA2 play in SSV9 infection remains to be explained. Since without PilA1 or PilA2 the cell is resistant to SSV9, we hypothesize that adsorption may be a two‐receptor process. For instance, in adsorption of bacteriophage T5 to *Escherichia coli*, a primary receptor reversibly binds O‐antigen on the LPS of the bacteria followed by a secondary irreversible interaction with FhuA leading to the injection of the phage DNA (Heller & Braun, 1982). A similar multi‐step process could be taking place here, where SSV9 interacts with a primary receptor prior to interacting with PilA1 and/or PilA2. Of the viruses tested, SSV13 was an exception and was able to infect all strains. This was surprising since the tail fibers of SSV9 and SSV13 encode identical protein sequences (data not shown). This suggests that at least one other protein is involved in SSV13 infection.

SIRV2 studies show physical interaction between virus and *aap* pili (Deng et al., 2014; Quemin et al., 2013). Interestingly, no mutations in the *aap* biosynthesis machinery were found in this study and the deletion of the *pilA1* and *pilA2* are sufficient for resistance to SIRV8. This difference between related virus resistance in related *Sulfolobus* strains could represent divergence in host specificity between SIRV2 and SIRV8. In addition, the lack of mutations in the biosynthesis machinery could be due to the genome organization of *S. islandicus* as compared to the *aap* locus to the characterized system in *S. acidocaldarius* (Figure S6)*.* The close proximity and high sequence identity of *pilA1* and *pilA2* genes encoded on opposite strands may make the deletion more favorable than the gene organization of related *Sulfolobus.* In the *S. acidocaldarius aap* locus, the *aap* pili are both encoded on the positive strand, and the structural components and a tRNA gene are found within the same gene cluster between the two pili genes (Henche, Ghosh, et al., 2012). In *S. solfataricus* P2*,* the pilin sequence homologs are distant from one another, are not in the same gene cluster as the predicted pilus machinery, and were previously predicted not to have *aap* (Henche, Ghosh, et al., 2012). Therefore, the gene localization of both *S. acidocaldarius* and *S. solfataricus* makes the deletion of both pilins less probable since it would be necessary to either delete several functional genes or involve multiple mutations. Furthermore, the deletion of both *pilA1* and *pilA2* may be favored if the distal pili biosynthesis machinery plays additional roles in *S. islandicus*. Therefore, we suspect that the difference in gene organization and role of the biosynthesis machinery may play a role in evolved viral resistance between the organisms. These species may employ different strategies to overcome viral predation, leading to the observed *pilA1* and *pilA2* deletions in *S. islandicus* in contrast with the *aap* biosynthesis machinery disruptions seen with SIRV2 in *S. solfataricus* P2.

Comparison between sequenced *Sulfolobus* genomes within the same environment and from around the globe reveals the maintenance of two pilin copies per genome (Figures 9 and S1). This finding combined with evidence of diversification within the global population suggests that there is pressure in nature to maintain two copies and develop CRISPR‐Cas immunity as a means to prevent subsequent infection (Figure S1). This may be related to PilA1 and PilaA2 adhesion function. Adhesion may play a role in niche establishment and biofilm formation, or a yet to be characterized natural function that is hijacked by the virus for attachment or entry. Therefore, while we are able to observe pilin deletions in the laboratory environment, in nature the host is under pressure to maintain the pilins and evade viral predation, which leads to a dynamic evolutionary virus–host relationship.

Virus–host dynamics vary dramatically across time and space in the highly structured populations of *S. islandicus* (Held, Herrera, Cadillo‐Quiroz, & Whitaker, 2010)*.* Temporal and spatial differences in virus–host interactions are most apparent in the highly studied populations of Kamchatka, Russia and Yellowstone National Park, USA (Pauly et al., 2019). Signatures recorded in the CRISPR‐Cas loci establish that the dominant CRISPR targeted viruses in Kamchatka are SSVs, while in Yellowstone National Park SIRVs are most targeted although SSVs are present in both environments (Pauly et al., 2019). Here we identified a surface structure encoded by two genes that is required for SSV9 infection in *S. islandicus* from Kamchatka, Russia and found that *pilA1* and *pilA2* were not only important for infection by SSVs isolated from Kamchatka, but also SIRVs isolated from Yellowstone National Park, USA. The hot spring environment contains many hosts and viruses evolving together and through the mechanistic analysis of these virus–host interactions, experimentation can infer how viral infection drives ecological patterns.

## 4 MATERIALS AND METHODS

### 4.1 Strains and growth conditions

*S. islandicus* strains were grown in dextrin tryptone (DT) media at pH 3.5 as described previously and supplemented with 20 μg/ml uracil (U), as needed (Whitaker, Grogan, & Taylor, 2003). All cultures were incubated in tissue culture flasks (Falcon; BD, United States) between 75 and 78°C without shaking. Solid plates were made with prewarmed 2× DT or 2× DTU supplemented with 20 mM MgSO_4_ and 7 mM CaCl_2_•2H_2_O and added in equal volume to 1.8% Gelrite and poured into Petri dishes. *E. coli* (Turbo DH5α, NEB) for molecular cloning plasmid construction was grown in Luria–Bertani medium at 37°C with added ampicillin (100 μg/ml was added when appropriate. Primers are listed in Table S4.

### 4.2 Virus preparation

SSV9 was isolated as previously described (Bautista et al., 2015). Chronically infected *S. islandicus* strain, G.V.10.6, was grown in DT media (described above) and SSV9 particles were isolated by removing the cells and collecting the filtrate from a 0.22‐μm polyethersulfone membrane filter (Millipore), when the density of the culture reached the mid‐log phase between OD_600_ = 0.15–0.2. SSV9 was stored until use in the dark at 4°C.

### 4.3 Resistant strain isolation and strain construction

*S. islandicus* SSV9‐resistant strains were performed in liquid medium in mid‐log phase *S. islandicus* cells grown (optical density OD_600_ between 0.09 and 0.15). Approximately 5.0 × 10^9^ cells were pelleted by centrifugation for 15 min at 4,000× *g* and the supernatant was decanted. Pellets were resuspended in 2 ml of either fresh media or live viral supernatants to an MOI of about 0.01 PFU/ml in 70 ml. The cultures were allowed to incubate for a week at 75 °C, where the enriched cultures were plated on solid media and incubated at 75°C for 10–14 days to isolate individual resistant strains. The resulting colonies were screened for chronic infection by amplifying the SSV9 capsid gene *vp2* (Table S4) and resistance was confirmed by spot‐on‐lawn tests as described previously (Bautista et al., 2015).

### 4.4 Genome sequencing

Genomic DNA from the evolved resistant strains was isolated as described previously (Whitaker et al., 2003). Genomic libraries were constructed using Nextera XT kit (Illumina) and sequenced using by W. M. Keck Center for Comparative and Functional Genomics at the University of Illinois, Urbana‐Champaign. Mutations were determined using breseq, comparing the reads from RJW002 to the evolved resistant strains (Deatherage & Barrick, 2014).

### 4.5 Genetic manipulation and shuttle vector complementation of *S. islandicus*

Recreation of the 6068 bp deletion was done via a Plasmid Integration and Segregation method as described previously (Zhang et al., 2013; Zhang & Whitaker, 2012). Complementation was performed by reintroducing regions into a shuttle vector pSeSD that was manipulated in *E. coli* and then transformed into *Sulfolobus* and maintained by uracil selection (Peng, Deng et al., 2012; Zhang et al., 2013; Zhang & Whitaker, 2012). Disruption of the *pibD* gene was performed via a microhomology‐mediated gene inactivation approach (MMGI) inserting the *argD* gene from *Sulfolobus tokodaii*, as was done previously (Zhang & Whitaker, 2018). A list of all primers used can be found in Table S4.

### 4.6 Virus PFU quantification

Plaque‐forming assays were performed to calculate the viral titer by incubating 100 μl of virus (10^–0^, 10^–1^ and 10^–2^ dilutions) with 10× concentrated host, *S. islancidus* strain Y.08.82.36, for 30 min at 75°C before plating in an overlay of Sucrose Yeast (SY) media and Gelrite (Redder et al., 2009). Plates were incubated 2–3 days until plaques were visible to enumerate and PFUs determined.

### 4.7 SSV9 qPCR quantification

Quantification of SSV9 genomes was determined by qPCR using primers UnvSSV 7F and UnvSSV 8F (Table S3) designed to amplify a 138‐bp section of the *vp1* coat gene. Each primer at 3 pmol was added to a reaction with 5 μl of SsoFast EvaGreen supermix (Bio‐Rad) and 0.5 μl of sample, and the volume was adjusted to 10 μl with PCR‐grade water. Three technical replicates were performed per sample in a Realplex (Eppendorf) thermocycler with the following protocol: 98°C for 2 min, 40 cycles of 98°C for 5 s followed by 60°C for 20 s. The standard curve was generated using a known amount of plasmid containing the target sequence (Bautista et al., 2015).

### 4.8 Screening for viral infection by the spot on lawn tests

Virus spots of 10 μl were placed on the host overlaid SY plates on three triplicate plates. Host was grown to mid‐log phase and concentrated 10‐fold in the overlay. Spotted plates were allowed to incubate 72 hr at 75°C and then examined for the presence or absence of a zone of inhibition. When testing for viral infection of a host, spots of the isolated culture's supernatant were tested on the susceptible host, Y08.82.36. The virus panel was created as described previously (Bautista et al., 2015). Virus stocks PFUs were normalized to 10^–3^ for SSVs and 10^–4^ for SIRVs and plated in an overlay of SY plates.

### 4.9 Adsorption assay and constant calculation

Two ml of a 10^4^ PFU/ml of SSV9 stock or 10^8^ PFU ml‐1 SIRV8 of stock in Wheaton vials was set up in triplicate where the host (8.3 × 10^8^ cells) was added to all but the control to assay for viral decay. This resulted in a different MOI for SSV9 (0.01) and SIRV8 (1) where disparity reflects the differences in virus production from the original host supernatants. RJW002, *Sulfolobus acidocaldarius* DSM 639, ΔA1 and ΔA1Δ6068 were added to the virus to assay virus adsorption. Samples were collected before cells were added and at 0, 5, 10, 15, 20, 25, 30, 40, 50, 60, 120 and 300 min after the addition of the host. Infection was halted by centrifugation at 15,000× *g* for 5 min and the supernatant was collected and stored at 4°C. Unadsorbed SSV9 or SIRV8 particles were measured in a plaque assay by the addition of 200 μl of a 10^–1^ dilution of the supernatant added to 500 μl of mid‐log‐phase, 10× *S. islandicus* Y08.82.36 cells. Cells mixed with virus dilutions were plated on overlays of SY medium and incubated at 75°C for 48 hr (Schleper, Kubo, & Zillig, 1992). Dilutions were performed and plated in triplicate. Three independent experiments were performed.

### 4.10 Infection assay

*S. islandicus* strains were grown to mid‐log phase (optical density OD_600_ between 0.09 and 0.15). Amounts of 1.48 × 10^9^ cells were centrifuged at 5,000× *g* for 15 min and resuspended in 2 ml of fresh media. Of the resuspension, half was added to 20 ml of either fresh media or SSV9 supernatant to an MOI of 0.01. The infections were incubated at 75°C for 1 hr followed by two washes with fresh DT media to remove unadsorbed viral particles. The SSV9‐challenged cells were then plated on solid media and allowed to incubate for 2–3 days at 75°C until PFUs were observed.

### 4.11 Double‐blind transmission electron microscopy

Cells were grown as described above and negatively stained with 2% uranyl acetate on copper grids. Strains were screened for pili and archaellum appendages in a double‐blind assay where cultures were blinded, images were captured of individual cells, randomized by a colleague and then analyzed by EFR for surface appendages and finally unblinded. Images were captured on a Philips CM200 TEM with a digital image acquisition using a TVIPS 2k × 2k Peltier‐cooled CCD camera.

### 4.12 Sequence alignments and selection modeling

BLASTp (Altschul et al., 1997) was used to find sequence homologs for *M164_2742* and *M164_2746*. Alignments were made with MEGA and maximum‐likelihood trees were created in RAxML (Kumar, Stecher, & Tamura, 2016; Stamatakis, 2014). The CODEML package from PAML was used to determine positive selected residues by Bayes Empirical Bayes analysis (Yang, 2007).

## AUTHOR CONTRIBUTIONS

MAB evolved many resistant strains and did preliminary characterization. CZ created the clean deletion strain. EFR performed all other experiments and analyses. RJW aided with the analysis of the data. EFR and RJW wrote the manuscript.

## Supporting information

Supinfo

## Data Availability

The data that support the findings of this study are available from the corresponding author upon reasonable request.

## References

[mmi14435-bib-0001] Albers, S.‐V. , & Pohlschröder, M. (2009). Diversity of archaeal type IV pilin‐like structures. Extremophiles, 13, 403–410. 10.1007/s00792-009-0241-7 19347566

[mmi14435-bib-0002] Albers, S.‐V. , Szabó, Z. , & Driessen, A. J. M. (2003). Archaeal homolog of bacterial type IV prepilin signal peptidases with broad substrate specificity. Journal of Bacteriology, 185, 3918–3925. 10.1128/JB.185.13.3918-3925.2003 12813086 PMC161584

[mmi14435-bib-0003] Altschul, S. F. , Madden, T. L. , Schäffer, A. A. , Zhang, J. , Zhang, Z. , Miller, W. , & Lipman, D. J. (1997). Gapped BLAST and PSI‐BLAST: A new generation of protein database search programs. Nucleic Acids Research, 25, 3389–3402. 10.1093/nar/25.17.3389 9254694 PMC146917

[mmi14435-bib-0004] Bautista, M. A. , Black, J. A. , Youngblut, N. D. , & Whitaker, R. J. (2017). Differentiation and structure in *Sulfolobus* *islandicus* rod‐shaped virus populations. Viruses, 9, e02565-14 10.3390/v9050120 PMC5454432 28534836

[mmi14435-bib-0005] Bautista, M. A. , Zhang, C. , & Whitaker, R. J. (2015). Induced dormancy in the archaeon *Sulfolobus* *islandicus* . mBio, 6, e02565‐14 10.1128/mBio.02565-14 25827422 PMC4453537

[mmi14435-bib-0006] Bize, A. , Karlsson, E. A. , Ekefjard, K. , Quax, T. E. F. , Pina, M. , Prevost, M.‐C. , … Prangishvili, D. (2009). A unique virus release mechanism in the archaea. Proceedings of the National Academy of Sciences, 106, 11306–11311. 10.1073/pnas.0901238106 PMC2708744 19549825

[mmi14435-bib-0007] Bolduc, B. , Wirth, J. F. , Mazurie, A. , & Young, M. J. (2015). Viral assemblage composition in Yellowstone acidic hot springs assessed by network analysis. ISME Journal, 9, 2162–2177. 10.1038/ismej.2015.28 26125684 PMC4579470

[mmi14435-bib-0008] Cadillo‐Quiroz, H. , Didelot, X. , Held, N. L. , Herrera, A. , Darling, A. , Reno, M. L. , … Whitaker, R. J. (2012). Patterns of gene flow define species of thermophilic archaea. PLoS Biology, 10, e1001265 10.1371/journal.pbio.1001265 22363207 PMC3283564

[mmi14435-bib-0009] Deatherage, D. E. , & Barrick, J. E. (2014). Identification of mutations in laboratory‐evolved microbes from next‐generation sequencing data using breseq. Methods in Molecular Biology (Clifton, N.J.), 1151, 165–188. 10.1007/978-1-4939-0554-6_12 PMC4239701 24838886

[mmi14435-bib-0010] Deng, L. , He, F. , Bhoobalan‐Chitty, Y. , Martinez‐Alvarez, L. , Guo, Y. , & Peng, X. (2014). Unveiling cell surface and type IV secretion proteins responsible for archaeal rudivirus entry. Journal of Virology, 88, 10264–10268. 10.1128/JVI.01495-14 24965447 PMC4136359

[mmi14435-bib-0011] Esquivel, R. N. , & Pohlschroder, M. (2014). A conserved type IV pilin signal peptide H‐domain is critical for the post‐translational regulation of flagella‐dependent motility. Molecular Microbiology, 93, 494–504. 10.1111/mmi.12673 24945931

[mmi14435-bib-0012] Esquivel, R. N. , Xu, R. , & Pohlschroder, M. (2013). Novel archaeal adhesion pilins with a conserved N terminus. Journal of Bacteriology, 195, 3808–3818. 10.1128/JB.00572-13 23794623 PMC3754589

[mmi14435-bib-0013] Held, N. L. , Herrera, A. , Cadillo‐Quiroz, H. , & Whitaker, R. J. (2010). CRISPR associated diversity within a population of *Sulfolobus* *islandicus* . PLoS ONE, 5, e12988 10.1371/journal.pone.0012988 20927396 PMC2946923

[mmi14435-bib-0014] Heller, K. , & Braun, V. (1982). Polymannose O‐antigens of *Escherichia coli*, the binding sites for the reversible adsorption of bacteriophage T5+ via the L‐shaped tail fibers. Journal of Virology, 41, 222–227. 7045389 10.1128/jvi.41.1.222-227.1982 PMC256742

[mmi14435-bib-0015] Henche, A.‐L. , Ghosh, A. , Yu, X. , Jeske, T. , Egelman, E. , & Albers, S.‐V. (2012). Structure and function of the adhesive type IV pilus of *Sulfolobus acidocaldarius* . Environmental Microbiology, 14, 3188–3202. 23078543 10.1111/j.1462-2920.2012.02898.x PMC3977132

[mmi14435-bib-0016] Henche, A.‐L. , Koerdt, A. , Ghosh, A. , & Albers, S.‐V. (2012). Influence of cell surface structures on crenarchaeal biofilm formation using a thermostable green fluorescent protein. Environmental Microbiology, 14, 779–793. 10.1111/j.1462-2920.2011.02638.x 22059595

[mmi14435-bib-0017] Koerdt, A. , Gödeke, J. , Berger, J. , Thormann, K. M. , & Albers, S. V. (2010). Crenarchaeal biofilm formation under extreme conditions. PLoS ONE, 5(11), e14104. 21124788 10.1371/journal.pone.0014104 PMC2991349

[mmi14435-bib-0018] Kumar, S. , Stecher, G. , & Tamura, K. (2016). MEGA7: molecular evolutionary genetics analysis version 7.0 for bigger datasets. Molecular Biology and Evolution, 33, 1870–1874. 10.1093/molbev/msw054 27004904 PMC8210823

[mmi14435-bib-0019] Makarova, K. S. , Koonin, E. V. , & Albers, S.‐V. (2016). Diversity and evolution of type IV pili systems in archaea. Frontiers in Microbiology, 7, 667 10.3389/fmicb.2016.00667 27199977 PMC4858521

[mmi14435-bib-0020] Pauly, M. D. , Bautista, M. A. , Black, J. A. , & Whitaker, R. J. (2019). Diversified local CRISPR‐Cas immunity to viruses of *Sulfolobus islandicus* . Philosophical Transactions of the Royal Society B Biological Sciences, 374, 20180093. 10.1098/rstb.2018.0093 PMC6452263 30905292

[mmi14435-bib-0021] Peng, N. , Deng, L. , Mei, Y. , Jiang, D. , Hu, Y. , Awayez, M. , … She, Q. (2012). A synthetic arabinose-inducible promoter confers high levels of recombinant protein expression in hyperthermophilic archaeon Sulfolobus islandicus. Applied and Environmental Microbiology, 78(16), 5630–5637. 22660711 10.1128/AEM.00855-12 PMC3406144

[mmi14435-bib-0022] Peng, X. , Garrett, R. A. , & She, Q. (2012). Archaeal viruses—Novel, diverse and enigmatic. Science China Life Sciences, 55, 422–433. 10.1007/s11427-012-4325-8 22645086

[mmi14435-bib-0023] Pohlschroder, M. , & Albers, S.‐V . (2016). Editorial: Archaeal cell envelope and surface structures. Microbial Physiology and Metabolism, 6, 1515. 10.3389/fmicb.2015.01515 PMC4701933 26779169

[mmi14435-bib-0024] Prangishvili, D. , Bamford, D. H. , Forterre, P. , Iranzo, J. , Koonin, E. V. , & Krupovic, M. (2017). The enigmatic archaeal virosphere. Nature Reviews Microbiology, 15, 724–739. 10.1038/nrmicro.2017.125 29123227

[mmi14435-bib-0025] Prangishvili, D. , & Garrett, R. A. (2005). Viruses of hyperthermophilic *Crenarchaea* . Trends in Microbiology, 13, 535–542. 10.1016/j.tim.2005.08.013 16154357

[mmi14435-bib-0026] Quemin, E. R. J. , Chlanda, P. , Sachse, M. , Forterre, P. , Prangishvili, D. , & Krupovic, M . (2016). Eukaryotic‐like virus budding in archaea. mBio, 7, e01439-16. 27624130 10.1128/mBio.01439-16 PMC5021807

[mmi14435-bib-0027] Quemin, E. R. J. , Lucas, S. , Daum, B. , Quax, T. E. F. , Kuhlbrandt, W. , Forterre, P. , … Krupovic, M. (2013). First insights into the entry process of hyperthermophilic archaeal viruses. Journal of Virology, 87, 13379–13385. 10.1128/JVI.02742-13 24089554 PMC3838266

[mmi14435-bib-0028] Redder, P. , Peng, X. U. , Brügger, K. , Shah, S. A. , Roesch, F. , Greve, B. O. , … Prangishvili, D. (2009). Four newly isolated fuselloviruses from extreme geothermal environments reveal unusual morphologies and a possible interviral recombination mechanism. Environmental Microbiology, 11, 2849–2862. 10.1111/j.1462-2920.2009.02009.x 19638177

[mmi14435-bib-0029] Rice, G. , Stedman, K. , Snyder, J. , Wiedenheft, B. , Willits, D. , Brumfield, S. , … Young, M. J. (2001). Viruses from extreme thermal environments. Proceedings of the National Academy of Sciences, 98, 13341–13345. 10.1073/pnas.231170198 PMC60872 11606757

[mmi14435-bib-0030] Schleper, C. , Kubo, K. , & Zillig, W. (1992). The particle SSV1 from the extremely thermophilic archaeon *Sulfolobus* is a virus: Demonstration of infectivity and of transfection with viral DNA. Proceedings of the National Academy of Sciences, 89, 7645–7649. 10.1073/pnas.89.16.7645 PMC49767 1502176

[mmi14435-bib-0031] Shahapure, R. , Driessen, R. P. C. , Haurat, M. F. , Albers, S.‐V. , & Dame, R. T. (2014). The archaellum: A rotating type IV pilus. Molecular Microbiology, 91, 716–723. 10.1111/mmi.12486 24330313

[mmi14435-bib-0032] Silverman, P. M. (1997). Towards a structural biology of bacterial conjugation. Molecular Microbiology, 23, 423–429. 10.1046/j.1365-2958.1997.2411604.x 9044277

[mmi14435-bib-0033] Snyder, J. C. , Bolduc, B. , & Young, M. J. (2015). 40 Years of archaeal virology: Expanding viral diversity. Virology, 479–480, 369–378. 10.1016/j.virol.2015.03.031 25866378

[mmi14435-bib-0034] Stamatakis, A. (2014). RAxML version 8: A tool for phylogenetic analysis and post‐analysis of large phylogenies. Bioinformatics, 30, 1312–1313. 10.1093/bioinformatics/btu033 24451623 PMC3998144

[mmi14435-bib-0035] Stedman, K. M. , DeYoung, M. , Saha, M. , Sherman, M. B. , & Morais, M. C. (2015). Structural insights into the architecture of the hyperthermophilic Fusellovirus SSV1. Virology, 474, 105–109. 10.1016/j.virol.2014.10.014 25463608

[mmi14435-bib-0036] Szabó, Z. , Sani, M. , Groeneveld, M. , Zolghadr, B. , Schelert, J. , Albers, S.‐V. , … Driessen, A. J. M. (2007). Flagellar motility and structure in the hyperthermoacidophilic archaeon *Sulfolobus* *solfataricus* . Journal of Bacteriology, 189, 4305–4309. 10.1128/JB.00042-07 17416662 PMC1913377

[mmi14435-bib-0037] Szabó, Z. , Stahl, A. O. , Albers, S.‐V. , Kissinger, J. C. , Driessen, A. J. M. , & Pohlschröder, M. (2007). Identification of diverse archaeal proteins with class III signal peptides cleaved by distinct archaeal prepilin peptidases. Journal of Bacteriology, 189, 772–778. 10.1128/JB.01547-06 17114255 PMC1797317

[mmi14435-bib-0038] Veith, A. , Klingl, A. , Zolghadr, B. , Lauber, K. , Mentele, R. , Lottspeich, F. *…* Kletzin, A . (2009). *Acidianus*, *Sulfolobus* and *Metallosphaera* surface layers: Structure, composition and gene expression. Molecular Microbiology, 73, 58–72. 19522740 10.1111/j.1365-2958.2009.06746.x

[mmi14435-bib-0039] Wang, F. , Cvirkaite‐Krupovic, V. , Kreutzberger, M. A. B. , Su, Z. , de Oliveira, G. A. P. , Osinski, T. , … Egelman, E. H. (2019). An extensively glycosylated archaeal pilus survives extreme conditions. Nature Microbiology, 1, 1401–1410. 10.1038/s41564-019-0458-x PMC6656605 31110358

[mmi14435-bib-0040] Whitaker, R. J. , Grogan, D. W. , & Taylor, J. W. (2003). Geographic barriers isolate endemic populations of hyperthermophilic archaea. Science, 301, 976–978. 10.1126/science.1086909 12881573

[mmi14435-bib-0041] Yang, Z. (2007). PAML 4: Phylogenetic analysis by maximum likelihood. Molecular Biology and Evolution, 24, 1586–1591. 10.1093/molbev/msm088 17483113

[mmi14435-bib-0042] Zhang, C. , Cooper, T. E. , Krause, D. J. , & Whitaker, R. J. (2013). Augmenting the genetic toolbox for *Sulfolobus islandicus* with a stringent positive selectable marker for agmatine prototrophy. Applied and Environment Microbiology, 79, 5539–5549. 10.1128/AEM.01608-13 PMC3754178 23835176

[mmi14435-bib-0043] Zhang, C. , Phillips, A. P. R. , Wipfler, R. L. , Olsen, G. J. , & Whitaker, R. J. (2018). The essential genome of the crenarchaeal model *Sulfolobus islandicus* . Nature Communications, 9, 4908 10.1038/s41467-018-07379-4 PMC6249222 30464174

[mmi14435-bib-0044] Zhang, C. , & Whitaker, R. J. (2012). A broadly applicable gene knockout system for the thermoacidophilic archaeon *Sulfolobus islandicus* based on simvastatin selection. Microbiology, 158, 1513–1522. 10.1099/mic.0.058289-0 22461488

[mmi14435-bib-0045] Zhang, C. , & Whitaker, R. J. (2018). Microhomology‐mediated high‐throughput gene inactivation strategy for the hyperthermophilic crenarchaeon *Sulfolobus* *islandicus* . Applied and Environment Microbiology, 84, e02167‐17. 10.1128/AEM.02167-17 PMC5734048 29030445

[mmi14435-bib-0046] Zhang, C. , Wipfler, R. L. , Li, Y. , Wang, Z. , Hallett, E. N. , & Whitaker, R. J . (2019). Cell structure changes in the hyperthermophilic crenarchaeon *Sulfolobus* *islandicus* lacking the S‐layer. mBio, 10, e01589-19 Retrieved from https://mbio.asm.org/content/10/4/e01589-19 31455649 10.1128/mBio.01589-19 PMC6712394

[mmi14435-bib-0047] Zink, I. A. , Pfeifer, K. , Wimmer, E. , Sleytr, U. B. , Schuster, B. , & Schleper, C. (2019). CRISPR‐mediated gene silencing reveals involvement of the archaeal S‐layer in cell division and virus infection. Nature Communications, 10, 1–14. 10.1038/s41467-019-12745-x PMC6805947 31641111

[mmi14435-bib-0048] Zolghadr, B. , Klingl, A. , Koerdt, A. , Driessen, A. J. M. , Rachel, R. , & Albers, S.‐V. (2010). Appendage‐mediated surface adherence of *Sulfolobus* *solfataricus* . Journal of Bacteriology, 192, 104–110. 10.1128/JB.01061-09 19854908 PMC2798249

[mmi14435-bib-0049] Zolghadr, B. , Klingl, A. , Rachel, R. , Driessen, A. J. M. , & Albers, S.‐V. (2011). The bindosome is a structural component of the *Sulfolobus solfataricus* cell envelope. Extremophiles, 15, 235–244. 10.1007/s00792-010-0353-0 21234771 PMC3047682

