# Supplementary material for: Surface resistance to SSVs and SIRVs in pilin deletions of Sulfolobus islandicus
Source: Mol Microbiol. 2019 Dec 19;113(4):718–27. doi: 10.1111/mmi.14435 (PMC7217056; doi:10.1111/mmi.14435)

Supplementary Table 1: **Experimentally evolved SSV9-resistant isolates.**

Parental Strain				Derived-resistant strains			
Strain	Genotype/Description	Phenotype	Reference	Experiment	Total Isolated	SSV9-infected	SSV9-uninfected
**∆A1**	∆pyrEF∆A1, CRISPR A1 repeat-spacer array deletion from RJW002	SSV9-susceptible, CRISPR spacer deletion	Bautista et al. 2015	1	96	92 (96%)	4 (4%)
				2	96	95 (99%)	1 (1%)
				3	40	38 (95%)	2 (5%)
				4	96	94 (98%)	2 (2%)

Supplementary Table 2. **Evolved SSV9-resistant isolates contain chromosomal mutations**.

Strain	Position	Mutation	Gene	SSV9 Susceptibility
**ΔA1.F6**	616743	C→T	M164_0673	-
	2,487,482	44 bp x 2	*M164_2678*	
	2,548,410	Δ6,068 bp	*[M164_2739]–[M164_2747]*	
**ΔA1.2310**	2,551,538	Δ2,310 bp	*[M164_2742]–[M164_2746]*	-
**ΔA1.3906**	2550354	Δ3,906 bp	*[M164_2740]–M164_2746*	-
**ΔA1.4609**	2,549,652	Δ4,609 bp	*[M164_2739]–M164_2746*	-

Supplementary Table 3. **Clean 6068bp deletion in ΔA1.**

Strain	Position	Mutation	Gene	SSV9 Susceptibility
**ΔA1Δ6068**	2,548,410	Δ6,068 bp	*[M164_2739]–[M164_2747]*	-

TABLE S4. List of Primers used in this study

Primers	Sequence (5'-3')
Full 6068 F	ATTAGTCGACACGAGAAGTGCCCCAACTAC
Full 6068 R	TAATGCTAGCTAAGCGATCCCTAAGCCACG
M164_2746_up.112.Sal_F	CGCGGTCGACAGTCTCCATGGTATTTAAT
M164_2746_Not.R	ATTGGCGGCCGCTTAGCTCGTTACTATTG
M164_2742_up.66.Sal_F	CGCGGTCGACCTATGTCATACTCAAGCA
M164_2742_Not.R	ATTGGCGGCCGCTCAGCTCGTTACTATTG
M164.2002_stoArgD_Fv2	GATTATTTTAAATCATGGGGTATCTAGTCCCTTGACAGCCGGATATGTGCTTTCATAT
M164.2002_stoArgD_Rv2	AATTAAAGGCTCTAGACCTATAATCGAGAAACGACTCGGGTCTATGGATTCATAGAC
M164.2002_stoArgD_Fv3	GCTGGTTCACACTTCAGTCCTTGATCTTAAATATAGAGAAGGATATGTGCTTTCATAT
M164.2002_stoArgD_rv3	TGGAGAAGGGGAGATTTCTAACATATCTATAGTACTTTATGTCTATGGATTCATAGAC
M164.2002_KO.seq.F	TTGATCTTCAGATAAGGAGAGTCAG
M164.2002_KO.seq.R	CCGTCTTCCTTTATCTCAGTC
6068_Dn.F	CGGGGTACCTATACTACGAGACATAGATATAGGCT
6068_Dn.R	AAGCGTCGACATCCTCACTCCTTAAAGCAAC
6068_Up.F	AAAACGCGTTAAGACCAGAAGTTTCCCTAAC
6068_Up.R	CGGGGTACCATGGTATACAATACGTTGTTACTACT
UnvSSV 7F	ATTCAGATTCTGWATWCAGAA
UnvSSV 8R	TCSCCTAACGCACTCATC

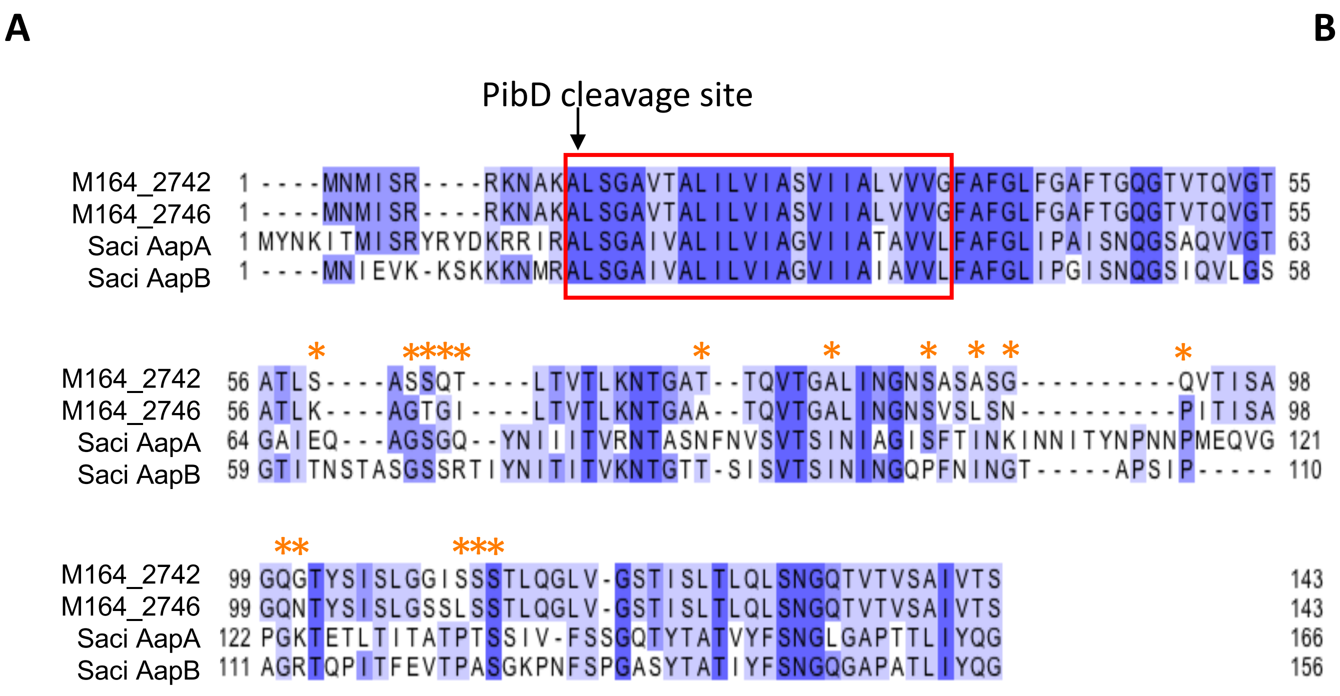

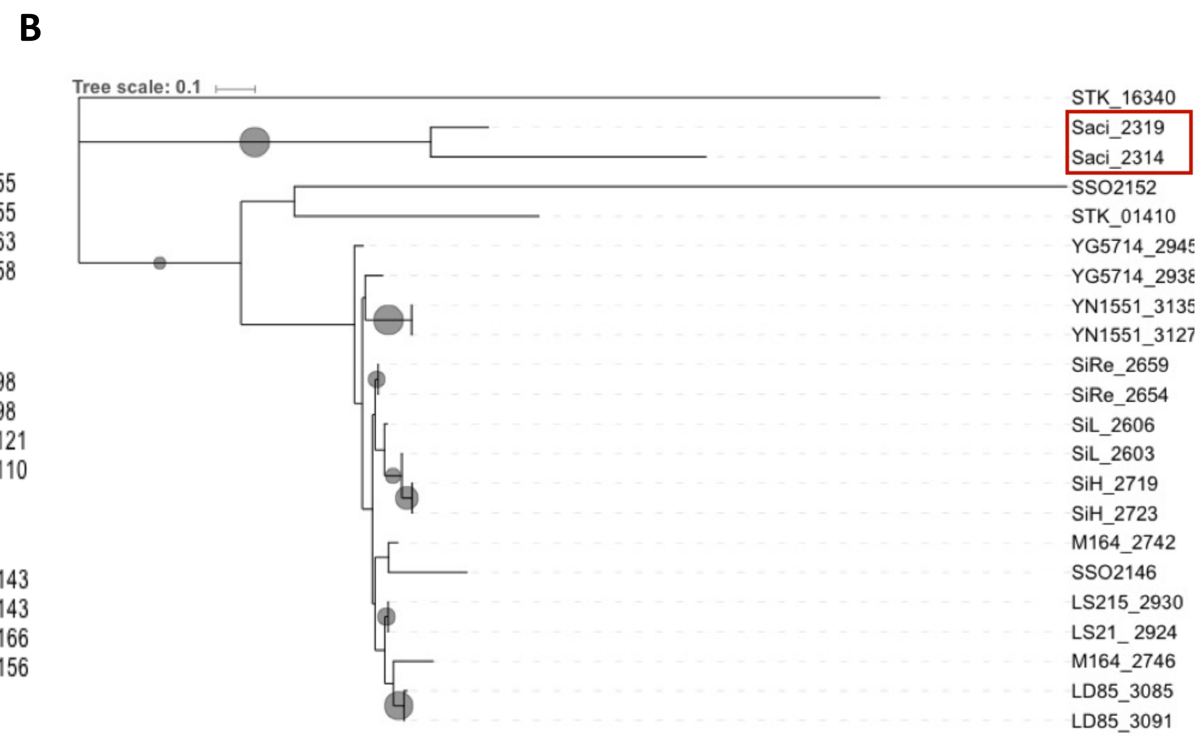

Figure S1. **Pilin homologs are undergoing positive selection. A.** Alignment of *S. islandicus* M164_2742 and M164_2746 with *S. acidocaldarius* AapA and AapB. Color coding is highlighted based on percent identity between *S. islandicus* and *S. acidocaldarius*. Conserved hydrophobic region (H-box) is highlighted in the red box (Esquivel et al., 2013). Pre-pilin peptidase cleavage point is denoted with the arrow. Positively selected residues in all of the *Sulfolobus* pilins shown in B are highlighted with orange asterisks and were determined by Bayes Empirical Bayes (BEB) analysis using in the codeml package of PAML (Yang, 2007). **B.** *Sulfolobus* homologs of putative pilin, M164_2742, are represented in a maximum-likelihood tree in RAxML (Stamatakis 2014). Bootstraps greater than 70 are proportionally shown in gray circles Two genes were identified where each strain is represented by a different color. Characterized archaeal adhesive pilus homologs, boxed in red, are identified in *S. acidocaldarius* (Henche *et al. 2012b).*

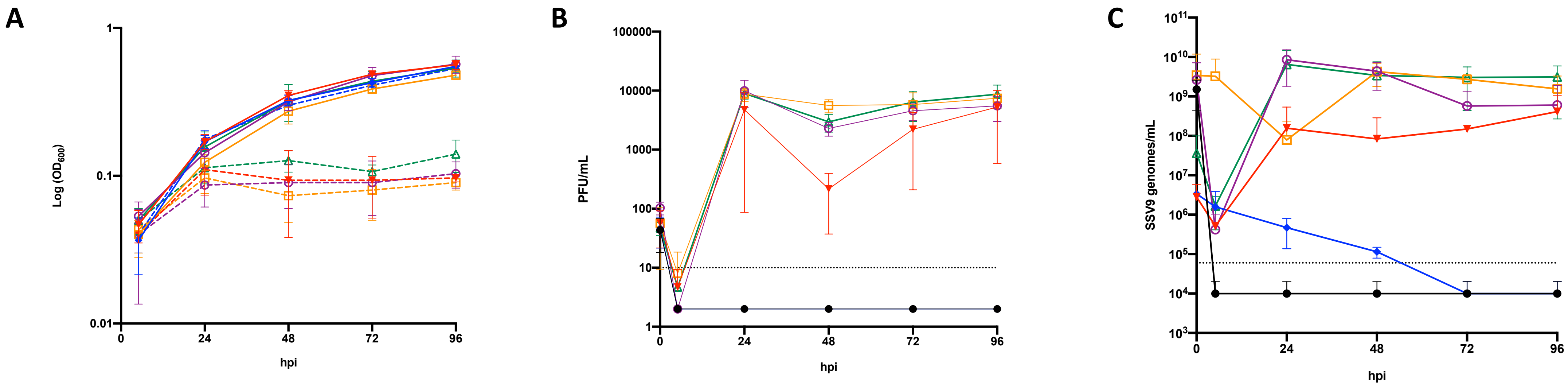

Figure S2. **Complements restore susceptibility of ΔA1Δ6068 . A.** Host growth (OD_{600}), **B.** PFU/mL, and **C.** genomes/mL over the course of SSV9 infection in in ΔA1 (red), ΔA1Δ6068 (blue), ΔA1Δ6068 pSeSD_2742 (purple), ΔA1Δ6068 pSeSD_2746 (orange). and ΔA1Δ6068 pSeSD_2741…2746 (green). In B and C, SSV9 decay over the course of infection is represented (black).

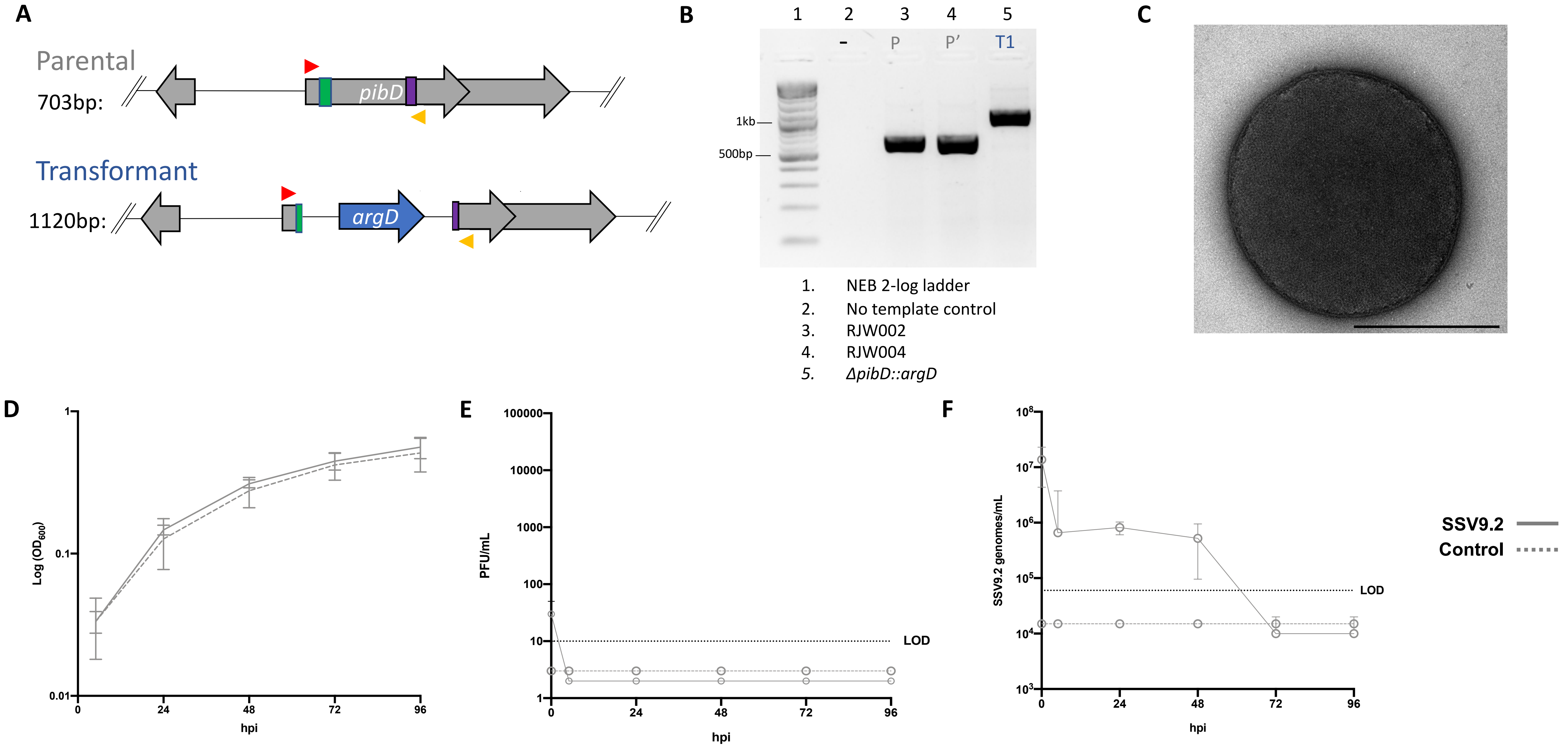

Figure S3. **Δ*pibD::argD* does not possess pilins on the surface resulting in SSV9.2 resistance .** **A.** Sematic of *pibD* disruption. Arrows are primers testing the transformation confirming disruption shown in **B.** **C.** TEM images of the resulting strain *ΔpibD::argD*. Scale bar = 1μm. **D-F.** Liquid infection of *ΔpibD::argD* with SSV9.2 at an MOI = 0.01.Solid lines are *ΔpibD::argD* challenged with SSV9.2 and the control shown in dashed lines. **D.** OD600. **E.** SSV9.2 PFU/mL **F.** SSv9.2 genomes/mL.

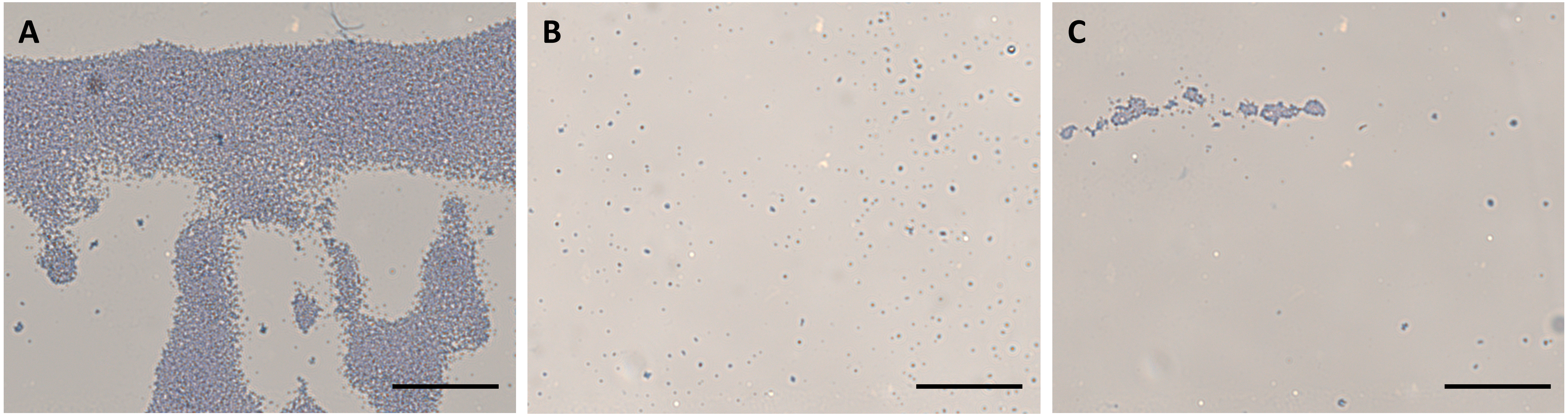

Figure S4. **ΔA1Δ6068 poorly adheres to a glass coverslip surface compared to RJW002.** Bright field microscopy at 40x magnification. Surface adhesion of crystal violet stained **A:** RJW002, **B:** *ΔpibD::argD*, **C:** ΔA1Δ6068 resistant strains assayed by an air liquid interface assay on a glass coverslip.

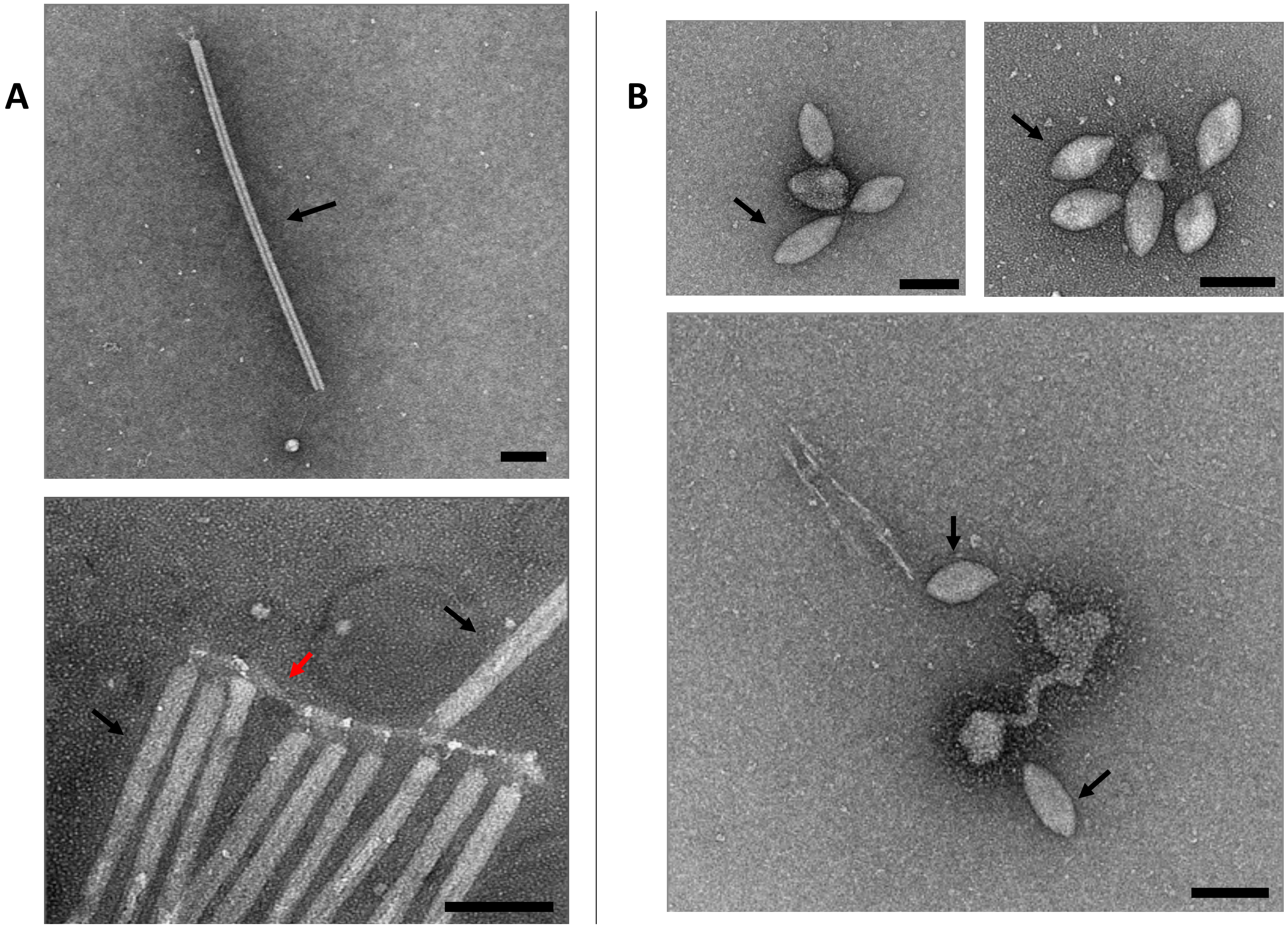

Figure S5. **A** and **B**: *S. islandicus Δcas6* (Bautista et. al. 2015) host supernatant was mixed with **A.** SIRV8 or **B.** SSV9. The host supernatants were filtered and the *Δcas6* and SSV9 supernatants were concentrated 20x in a 30kDa membrane filter. Host and virus supernatants were mixed at an approximate MOI of 1 and incubated at 75ºC for an hour before imaging under TEM. Black arrows are pointing to virus, red arrows are pointing to pili. Scale bars are 100nm.

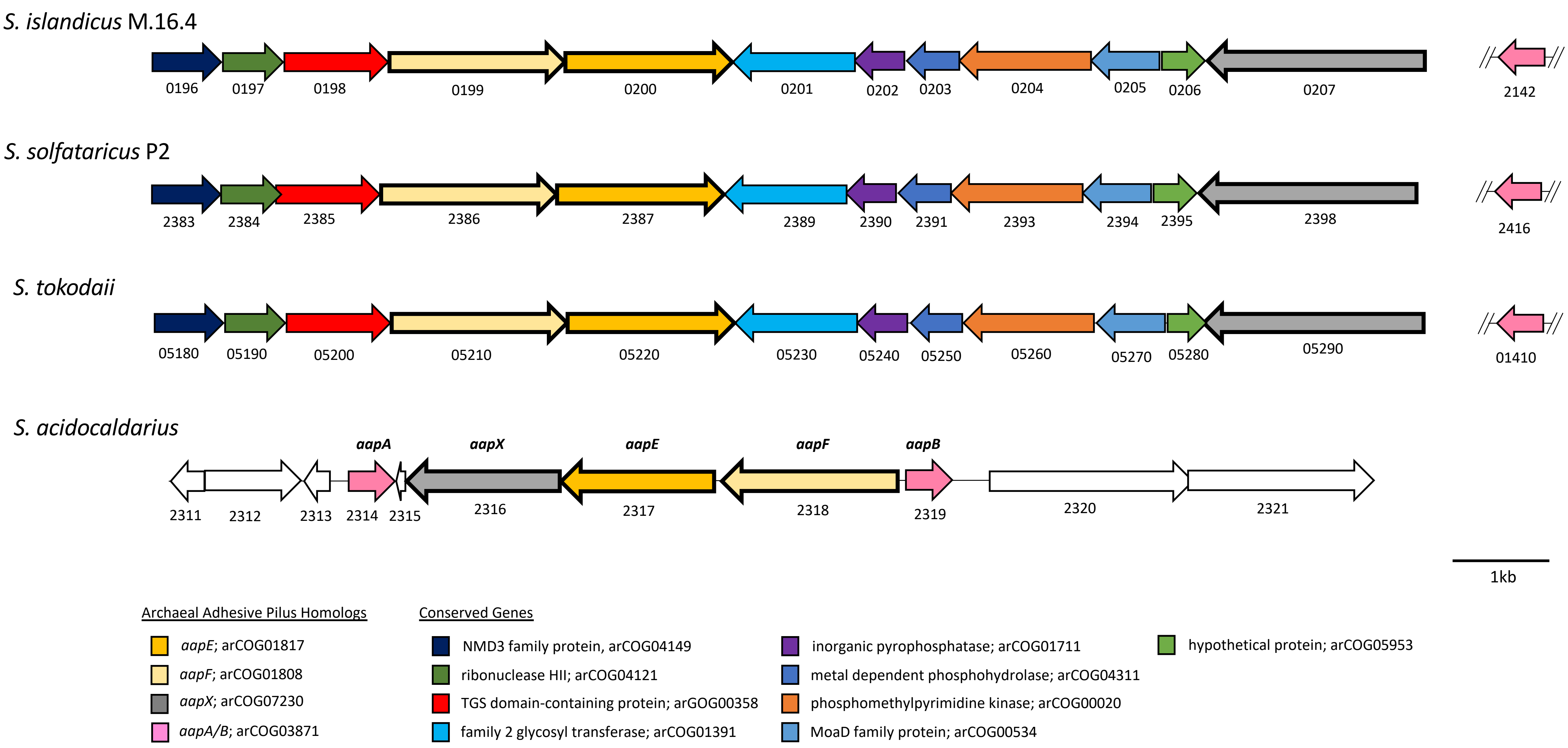

Supplementary Figure 6: Pilin machinery in representative *Sulfolobus* genomes (bolded arrows) compared to the characterized archaeal adhesive pilus in *Sulfolobus acidocaldarius* (Henche at al. 2012a). The percent protein blast match compared to the *S. acidocaldarius* is highlighted under each of the essential genes machinery for pilin filament production.

SSV9.2 Supplement

<u>SSV9.2: CRISPR-Cas immunity escape virus:</u>

CRISPR-Cas evading SSV9 variant (SSV9.2) isolation.
Culture was prepared as described in the resistant strain isolation method above where the CRISPR-Cas immune strain was mixed with SSV9 supernatant at an MOI of 0.01. The resulting strain, Δ*cas6*::SSV9.2, was sequenced to reveal a chronically infected strain with an SSV9 variant that contained a 6872bp chromosomal deletion. This deletion in SSV9 removed the CRISPR-Cas spacer match immunity conferred in RJW002 and RJW004, thereby rendering these strains susceptible to SSV9.2.

SSV9.2 Genome Map
The viral genome deletion of 6872bp spans 2822..9693bp and is represented in the SSV9 genome below map in blue. The deletion disrupts the integrase and deletes an additional 8 viral ORFS (gp09 to gp16). Deletion of the 100% protospacer match with a PAM encoded by the native host, M.16.4, is represented in orange (9460..9599bp).

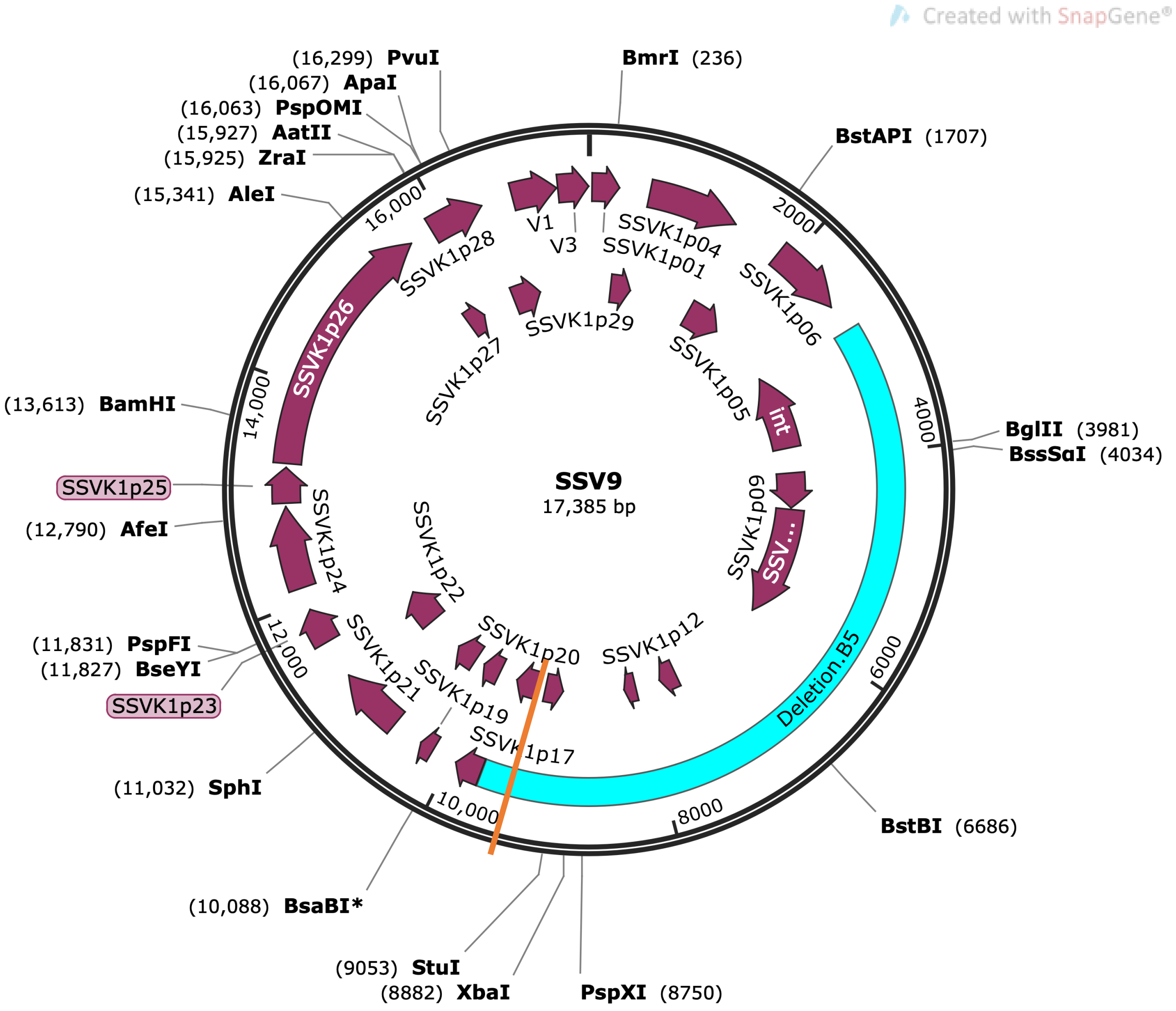

Supplement: Supplementary file 1 — Supinfo [file MMI-113-718-s001.pdf]
